# The effect of Tai Chi on glycemic control in type 2 diabetes mellitus: a meta-analysis of randomized controlled trials

**DOI:** 10.3389/fendo.2025.1605253

**Published:** 2025-08-12

**Authors:** Yawei Sun, Quanzhi Li, Weiqi Xue

**Affiliations:** ^1^ Sports Department, Nanjing Agricultural University, Nanjing, China; ^2^ Sports Training Academy, Nanjing Institute of Physical Education and Sports, Nanjing, China

**Keywords:** Tai Chi, T2DM, HbA1c, blood lipids, inflammatory markers, meta-analysis

## Abstract

**Objective:**

This study aimed to investigate the effects of Tai Chi on fasting blood glucose (FBG), HbA1c, blood lipids, blood pressure, and inflammatory markers in patients with type 2 diabetes mellitus (T2DM) through a meta-analysis.

**Methods:**

We systematically searched PubMed, Cochrane Library, CNKI, CBM, WanFang, Web of Science, and Embase databases for randomized controlled trials (RCTs) evaluating Tai Chi interventions in T2DM patients. Statistical analyses were performed using Review Manager 5.4 software with random-effects models.

**Results:**

Compared to the control group, Tai Chi significantly reduced: FBG(SMD= -0.57, 95% CI [-0.92,-0.23], *P* =0.001), HbA1c(MD=-0.73, 95%CI[-0.98, -0.49], *P*<0.00001), TG(SMD=-0.50, 95%CI[-0.91, -0.09], *P* =0.02), LDL-C(SMD=-0.70, 95%CI[-1.26, -0.15], *P* =0.01), hs-CRP(SMD=-0.71, 95%CI[-1.10, -0.31], *P* =0.0005), IL-6(SMD=-2.11, 95%CI[-2.88, -1.34], *P*<0.00001), TNF-α(SMD=-3.25, 95%CI[-3.25, -0.53], *P* =0.006). Subgroup analyses indicated optimal FBG reduction with: The standardized 24-form Tai Chi routine; Interventions ≥12 weeks in duration; Exercise frequency >5 sessions/week; Daily exercise duration ≥60 minutes.

**Conclusion:**

Tai Chi demonstrates clinically meaningful benefits for T2DM management. Future RCTs should explore age-specific (e.g., pediatric vs. geriatric) and sex-stratified responses to inform personalized exercise prescriptions.

## 1 Introduction

The global prevalence of diabetes continues to rise alongside increasing obesity rates, sedentary behaviors, and poor dietary patterns, establishing a critical public health challenge requiring urgent intervention (1, 2). Epidemiological surveillance by the World Health Organization (WHO) and International Diabetes Federation (IDF) identified 425 million diabetes cases globally in 2017, with 4 million annual deaths attributable to diabetes, representing 8.8% of global all-cause mortality (3, 4). As the third most prevalent chronic non-communicable disease, diabetes accounts for 9.6% of global disability-adjusted life years (DALYs) and is ranked fourth among contributors to the global disability burden (5). The economic ramifications are equally profound, with global diabetes-related expenditures reaching $966 billion USD in 2021 (6)—projected to consume 13% of international healthcare budgets by 2025 (7). Despite the therapeutic benefits of oral hypoglycemic agents and insulin therapy, these interventions carry risks including hypoglycemia, weight gain, insulin resistance, and complications ranging from gastrointestinal dysmotility (8), progressive retinopathy (9), and diabetic nephropathy (10), micronutrient imbalances, inflammatory cytokine elevation (11) and peripheral edema (12). Numerous clinical studies have found that obesity, caused by the accumulation of visceral fat, is a primary risk factor for type 2 diabetes mellitus (T2DM), disrupting glucose and lipid metabolism and leading to insulin resistance (13). Clinical trials demonstrate that each kilogram of weight loss correlates with a 16% reduction in T2DM risk (14). This evidence reveals a therapeutic paradox: although obesity elevates T2DM risk, many glucose-lowering therapies promote weight gain—whereas weight loss reduces T2DM incidence. Consequently, non-pharmacological interventions have become critical. The IDF and American Diabetes Association (ADA) now endorse aerobic exercise as a cornerstone strategy for T2DM management.

Tai Chi, as a traditional Chinese exercise and wellness method, combines physical movement, breathing techniques, and mental focus. It emphasizes a balanced development of both mind and body while aiming to maintain dynamic equilibrium. Tai Chi is widely accessible, especially for diabetic patients who can adjust the exercise intensity and duration according to their age and physical condition, which ensures personalization and suitability (15). It is noteworthy that vigorous exercise can increase the cardiovascular burden in patients with diabetes and obesity, as well as raise the incidence of hypoglycemia (16). In contrast, Tai Chi is popular in communities and healthcare settings because it is gentle, slow-paced, easy to learn, and technically undemanding, making it especially suitable for people with prediabetes (17). Furthermore, Tai Chi significantly improves balance, coordination, and muscle strength in older adults, thereby reducing the risk of falls (17). Therefore, we hypothesize that Tai Chi might serve as a universally applicable rehabilitation method for patients with T2DM.

A large body of evidence (18–22) suggests that high fasting blood glucose (FBG) levels damage pancreatic β-cell function, leading to insufficient insulin secretion and elevated blood glucose levels. HbA1c reflects the average blood glucose level over the past 2–3 months and is used to evaluate blood glucose control in patients with T2DM. Increased HbA1c levels indicate deteriorating pancreatic β-cell function, which may lead to progressive pancreatic dysfunction. Lipid metabolism disorders not only affect insulin sensitivity but furthermore worsen blood glucose control by promoting pancreatic β-cell apoptosis and insulin secretion dysfunction. The levels of fasting insulin (Fins), high-sensitivity C-reactive protein (hs-CRP), interleukin-6 (IL-6), and tumor necrosis factor-alpha (TNF-α) are positively correlated with glucose metabolism disorders and insulin resistance in patients with T2DM. These inflammatory markers further exacerbate insulin resistance by directly affecting β-cell function and insulin sensitivity. Studies have shown inconsistent conclusions regarding Tai Chi’s effect on improving FBG (23, 24); therefore, we propose that a meta-analysis is necessary. Zhao (25) conducted a meta-analysis on Tai Chi’s effects on FPG and HbA1c, demonstrating significant improvements; however, it did not analyze the impact of inflammatory markers on blood glucose control in T2DM. Similarly, Tang (26) emphasized the need to investigate different Tai Chi styles’ effects on blood glucose in T2DM patients, while existing studies often lack subgroup analyses based on exercise duration, intervention period, and frequency (27).

In summary, given the potential variations in the effects of Tai Chi types, exercise duration, intervention period, and frequency on FBG, HbA1c, blood lipids, inflammatory markers, and blood pressure in patients with T2DM, this study synthesizes existing research on Tai Chi’s role in blood glucose regulation among individuals with T2DM. We employ meta-analysis methods to conduct systematic, objective, and quantitative statistical analyses, thereby generating evidence to support the development of scientifically grounded exercise prescriptions.

## 2 Materials and methods

### 2.1 Search strategy

This study conducted a systematic search for randomized controlled trials (RCTs) investigating the effects of Tai Chi on blood glucose regulation in patients with T2DM across seven electronic databases, including PubMed, Cochrane Library, China National Knowledge Infrastructure (CNKI), Chinese Biomedical Database (CBM), WanFang, Web of Science, and Embase. Search terms encompassed “Tai Chi,” “Tai Chi Chuan,” “type 2 diabetes mellitus (T2DM),” “hyperglycemia,” “insulin,” “fasting blood glucose,” and “blood lipids,” with controlled vocabulary adapted to each database. No restrictions were applied to Boolean operator combinations of s The search period spanned from database inception through April 1, 2025. However, studies published prior to 2010 were excluded during screening (see Section 2.2.2) due to outdated diagnostic criteria for T2DM (ADA standards revised post-2010).Inclusion criteria focused exclusively on RCTs examining Tai Chi’s effects on blood glucose regulation in patients with T2DM, with no language restrictions imposed. The complete search strategy is detailed in **Table 1**.

**Table 1:** Search strategy.

Database	Search criteria
PubMed	#1 “Diabetes Mellitus”[Mesh] OR Type 2 Diabetes Mellitus [Title/Abstract] OR Type 2 diabetes [Title/Abstract] OR Blood Pressure [Title/Abstract] OR Fasting Blood Glucose [Title/Abstract] OR HbA1c [Title/Abstract] OR Glycosylated Hemoglobin ([Title/Abstract] OR Fasting Insulin [Title/Abstract] OR Blood Lipid [Title/Abstract] OR Inflammatory Cytokines[Title/Abstract]. #2 “Tai Ji” [Mesh] OR Tai Chi Chuan [Title/Abstract] OR Tai-ji [Title/Abstract] OR Tai Chi [Title/Abstract] OR Tai Ji Quan [Title/Abstract] OR Taiji [Title/Abstract] OR Taijiquan [Title/Abstract]. #3–1 AND 2.
Cochrane Library	#1 Mesh: [Tai Ji] explode all trees. #2 Mesh: [Diabetes Mellitus] explode all trees. #3 (Tai-ji): Title, Abstract, Keywords. #4 (Tai Chi): Title, Abstract, Keywords. #5 (Tai Chi Chuan): Title, Abstract, Keywords. #6 (Tai Ji Quan): Title, Abstract, Keywords. #7 (Taiji): Title, Abstract, Keywords. #8 (Taijiquan): Title, Abstract, Keywords. #9 (Tai Ji): Title, Abstract, Keywords. #10 (Type 2 Diabetes Mellitus): Title, Abstract, Keywords. #11 (Fasting Insulin): Title, Abstract, Keywords. #12 (Fasting Blood Glucose): Title, Abstract, Keywords. #13 (HbA1c): Title, Abstract, Keywords. #14 (Blood Lipid): Title, Abstract, Keywords. #15 (Inflammatory Cytokines): Title, Abstract, Keywords. #16 #1 OR #3 OR #4 OR #5 OR #6 OR #7 OR #8 OR #9. #17 #2 OR #10 OR #11 OR #12 OR #13 OR #14 OR #15. #18 #16 OR #17.
CNKI	#1 Mesh: tai ji (Tai Chi). #2 Mesh: High blood sugar or type 2 diabetes. #3 #1 and #2. #4 Title, Abstract, Keywords: tai ji (Tai Chi). #5 Title, Abstract, Keywords: High blood sugar or type 2 diabetes or insulin or FBG or lipids. #6 #4 and #5.
CBM	#1 Title: TAICHI or taiji. #2 Title: High blood sugar or type 2 diabetes or insulin or FBG or lipids. #3 #1 and #2. #4 Abstract: TAICHI or taiji. #5 Abstract: High blood sugar or type 2 diabetes or insulin or FBG or lipids. #6 #4 and #5.
WanFang	#1 Mesh: tai ji (Tai Chi). #2 Mesh: High blood sugar or type 2 diabetes or insulin or FBG or lipids. #3 #1 and #2 #4 Title/Abstract: tai ji (Tai Chi). #5 Title/Abstract: High blood sugar or type 2 diabetes or insulin or FBG or lipids. #6 #4 and #5。 #7 Abstract: tai ji (Tai Chi). #8 Abstract: High blood sugar or type 2 diabetes or insulin or FBG or lipids. #9 #7 and #8.
Web of Science	#1 Mesh: Tai-ji、Tai Chi、Tai Chi Chuan、Tai Ji Quan、Taiji、Taijiquan、Tai Ji。 #2 Mesh: Type 2 Diabetes Mellitus OR Type 2 Diabetes Mellitus。 #3 #1 and #2 #4 Title: Tai-ji、Tai Chi&、Tai Chi Chuan、Tai Ji Quan、Taiji、Taijiquan、Tai Ji。 #5 Title: Diabetes Mellitus OR Type 2 Diabetes Mellitus OR Blood Pressure OR Fasting Blood Glucose OR HbA1c OR Blood Lipid OR Inflammatory Cytokines。 #6 #4 and #5。 #7 Abstract: Tai-ji、Tai Chi、Tai Chi Chuan、Tai Ji Quan、Taiji、Taijiquan、Tai Ji。 #8 Abstract: Diabetes Mellitus OR Type 2 Diabetes Mellitus OR Blood Pressure OR Fasting Blood Glucose OR HbA1c OR Blood Lipid OR Inflammatory Cytokines。 #9 #7 and #8.
Embase	#1 ‘Tai Chi’/Expanded Thesaurus Terms #2 ‘tai ji’: abstract, title #3 ‘tai chi chuan’: abstract, title #4 ‘tai-ji’: abstract, title #5 ‘tai ji quan’: abstract, title #6 ‘taiji’: abstract, title #7 ‘taijiquan’: abstract, title #8 ‘Diabetes Mellitus’/Expanded Thesaurus Terms #9 ‘Type 2 Diabetes Mellitus’: abstract, title #10 ‘randomized controlled trial’/Expanded Thesaurus Terms #11 #1 OR #2 OR #3 OR #4 OR #5 OR #6 OR #7 #12 #8 OR #9 #13 #11 OR #12 #14 #10 AND #12 AND #13

### 2.2 Inclusion and exclusion criteria for literature

Inclusion and exclusion criteria were defined according to the PICOS framework (28).

#### 2.2.1 Inclusion criteria

(1) Study population: Patients(≥18 years) with T2DM were required to meet diagnostic criteria of the ADA, defined as FBG ≥126 mg/dL (7.0 mmol/L), random blood glucose ≥200 mg/dL (11.1 mmol/L), or glycated hemoglobin (HbA1c) ≥6.5%. Studies including male/female/both sexes were eligible. (2) Interventions: Experimental group: Structured Tai Chi interventions (e.g., Yang-style, Chen-style, or other standardized forms). Control group: Conventional treatments (e.g., pharmacotherapy, basic health education) or alternative physical exercises (e.g., jogging). (3) Outcome measures: Complete pre- and post-intervention data were required for the following metabolic parameters: FBG, HbA1c, total cholesterol (TC), low-density lipoprotein cholesterol (LDL-C), high-density lipoprotein cholesterol (HDL-C), triglycerides (TG), diastolic blood pressure (DBP), systolic blood pressure (SBP), insulin, and inflammatory markers. (4) Study Design: Only RCTs were eligible to ensure methodological rigor.

#### 2.2.2 Exclusion criteria

Study population: Exclusion criteria comprised secondary diabetes, severe cardiovascular diseases (e.g., acute myocardial infarction, heart failure), or cerebrovascular events (e.g., stroke). (2) Study design: Non-randomized controlled trials (non-RCTs) were excluded to maintain validity of evidence synthesis. Interventions: Participants receiving adjunct therapies (e.g., massage, traditional Chinese medicine, acupuncture) in either group were excluded. (4) Outcome Measures: Studies lacking complete data for primary outcomes (e.g., FBG, HbA1c) were excluded. (5) Data currency and duplication: Duplicate publications and studies published prior to 2010 were excluded to prioritize contemporaneous evidence.

### 2.3 Literature screening and data extraction

Two investigators (SYW and LQZ) independently performed literature screening and data extraction, with cross-checking at each stage. The workflow comprised the following steps: (1) Initial literature search: Implementation of the search strategy detailed in **Table 1**.(2) Deduplication: Retrieved records were imported into EndNote X9 using the ENW file format, followed by removal of duplicate entries.(3) Title/abstract screening: Secondary screening was conducted through independent review of titles and abstracts.(4) Full-text assessment: Eligible articles underwent full-text retrieval and critical appraisal, culminating in a tertiary screening round to confirm final inclusion. Discrepancies were resolved by adjudication from a third investigator (XWQ). Data extraction fields included: First author name, Publication year, Participant demographics (age range, sample size), Intervention characteristics (experimental/control group protocols, duration, frequency, session length)and outcome measures.

### 2.4 Literature quality assessment

Methodological quality was assessed using the Cochrane Risk of Bias Tool (RoB 2.0) within Review Manager 5.4 software (Cochrane Collaboration). The tool evaluates seven domains of bias risk: Random sequence generation, Allocation concealment, Blinding of participants and personnel, Blinding of outcome assessment, Incomplete outcome data, Selective reporting, Other sources of bias. Each domain was classified into three risk categories: low risk, high risk, or unclear risk. Results were synthesized into a risk-of-bias summary figure, generated directly through RevMan 5.4.

### 2.5 Data analysis

A systematic approach to data extraction and analysis was implemented, comprising the following steps:(1) Continuous variables (reported as mean ± standard deviation) were independently extracted by two investigators from included RCTs. (2)Statistical analysis: Analyses were conducted in RevMan 5.4 (Cochrane Collaboration), structured as follows: 1) Literature management: Study metadata (e.g., authorship, publication year) were catalogued in the “Studies and References” module to establish a structured literature repository.2) Risk of bias assessment: Methodological quality was appraised via the Cochrane RoB 2.0 tool through single-blind assessment by two independent reviewers.3) Data entry: Outcome measures from individual RCTs were systematically input into the “Data and Analysis module” using a predefined extraction template to ensure accuracy.4) Effect size calculation: Continuous variables with homogeneous units were analyzed via mean difference (MD) with 95% confidence intervals (CI). Variables with heterogeneous units or non-continuous scales were standardized using standardized mean difference (SMD) (29).

Heterogeneity quantification: Heterogeneity was quantified via the I² statistic, interpreted as follows (30): I²= 0%: No heterogeneity (fixed-effect model); 0% < I²≤ 50%: Low heterogeneity (fixed-effect model); 50%<I²≤75%: Moderate heterogeneity (random-effects model); I²> 75%: High heterogeneity (random-effects model with subgroup and sensitivity analyses).

### 2.6 Sensitivity analysis

Sensitivity analysis was conducted by iteratively excluding individual studies and re-running meta-analyses on the remaining dataset to assess result stability pre- and post-exclusion.

### 2.7 Subgroup analysis

Subgroup analyses of the included studies were stratified by Tai Chi style, intervention duration (weeks), weekly frequency, and Daily Exercise Duration (minutes).

## 3 Results

### 3.1 Literature screening results

**Figure 1:**
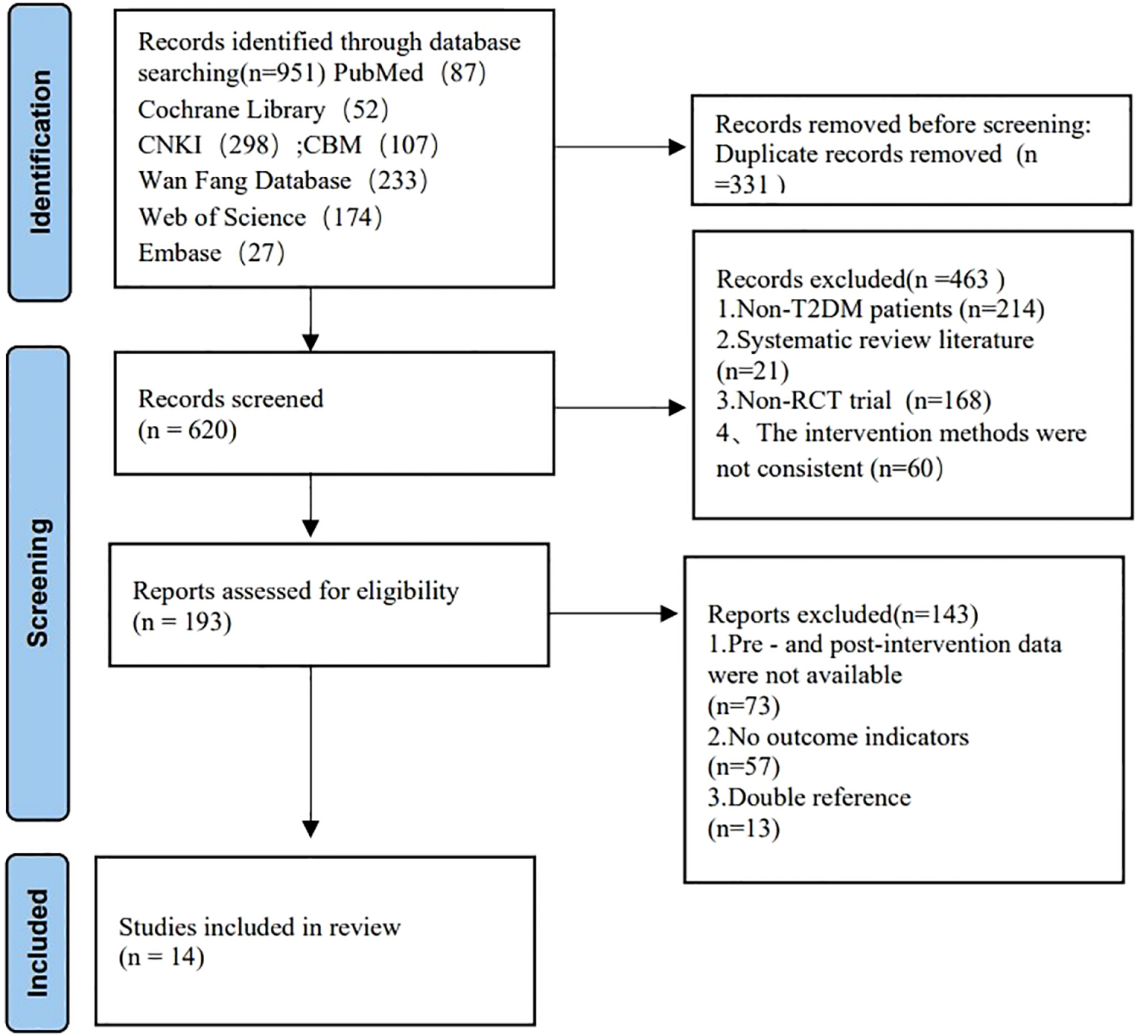
PRISMA flow diagram of literature screening.

The systematic search across six databases initially identified 951 records. 331 duplicates were removed through automated and manual deduplication. Title/abstract screening excluded 463 records for the following reasons: Non-target populations (e.g., Type 1 diabetes mellitus, gestational diabetes, secondary diabetes, other metabolic disorders); Non-RCT designs (e.g., reviews, commentaries, case reports, animal studies); Non-Tai Chi interventions (e.g., yoga, Baduanjin, pharmacotherapy-only, dietary interventions); Insufficient data (e.g., missing original data, non-quantitative outcomes). Full-text review excluded 143 articles due to: Absence of diabetes-specific outcomes; Combined interventions (e.g., pharmacotherapy, acupuncture) without isolated Tai Chi groups; Data incompleteness or methodological flaws (e.g., statistical errors). Final inclusion comprised 14 RCTs meeting all eligibility criteria. Grey literature (theses, conference proceedings) manually searched but yielded no additional eligible studies. The PRISMA flow diagram of literature screening in **Figure 1** (31).

### 3.2 Basic characteristics of the literature

Fourteen studies meeting the predefined eligibility criteria were selected, comprising 1,311 participants. Intervention styles: Tai Chi styles: 24-style Tai Chi (n = 6 studies), Yang-style (n = 3), Chen-style (n = 3), and unspecified styles (n = 2). Control group: Standard care (e.g., pharmacotherapy, dietary monitoring) or non-exercise controls. Intervention duration: 8–24 weeks. Complete intervention characteristics are tabulated in **Table 2**.

**Table 2:** Characteristics of included studies.

Author	Age (E/C)	Sample size(E/C)	Intervention(E)	Intervention(C)	Country	Language	Duration	Frequency	Daily exercise duration	Outcome indicators
Ahn (32)	66.05 ± 6.42/62.73 ± 7.53	20/19	Yang-style	Routine care	America	English	12W	2sessions/week	60min	1,2
Chan (33)	64.70 ± 7.59/65.13 ± 10.22	69/ 62	24-style	Routine care	England	English	12W	2sessions/week	60min	1,2,7,8,9,10,12
Chen (34)	59.1 ± 6.2/ 57.4 ± 5.8	56/ 48	Chen-style	Aerobics class	America	English	12W	3sessions/week	60min	1,2,9,10,11
Gao (23)	60.5 ± 6.8/61.4 ± 7.5	65/ 65	24-style	Routine care	China	Chinese	12W	7sessions/week	60 min	1,2,4
Hui (1)	45.9 ± 5.2/44.9 ± 5.6	124/129	Yang-style	No extra exercise	China	English	12W	5sessions/week	30min	1,7,8,9,10,11,12
Hu (35)	61.45 ± 4.97/59.55 ± 6.19	30/30	24-style	No extra exercise	China	Chinese	12W	4sessions/week	80min	1,2,3,4,7,8,9,10,11,12
Li (24)	62.91 ± 2.48/62.91± 2. 48	50/ 50	Chen-style	Regular exercise	China	Chinese	24W	7sessions/week	40–50 min	1,2,3,7,8,9,10,11,12
Liu (36)	60.6 ± 5.7/61.3 ± 4.9	16/ 16	Yang-style	No extra exercise	China	English	24W	6sessions/week	80min	1,2,3,5,6
Li (37)	57.3 ± 10.3	30/ 30	TAICHI	Routine care	China	Chinese	8W	7sessions/week	45min	1,3,4,5,6
Meng (38)	68. 4 ± 3. 2	100/ 100	TAICHI	Routine care	China	Chinese	12W	4sessions/week	30 min	1,2,3,9,10,11,12
Song (39)	54	40/ 40	24-style	Routine care	China	Chinese	12W	7sessions/week	50min	1,2
Zhao (13)	54. 75± 6. 09/54. 75± 6. 09	8/ 8	Chen-style	No extra exercise	China	Chinese	16W	7sessions/week	60min	1,9,10,11,12
Zhou (40)	62.12 ± 7.59/60.9 ± 6.99	33/ 33	24-style	Routine care	China	Chinese	24W	6sessions/week	60min	1,2,4,5,6
Zhu (41)	64. 5 ± 4. 3/64. 5± 4. 3	20/ 20	24-style	Routine care	China	Chinese	16W	4sessions/week	60min	1,9,10,11,12

E, Experimental group; C, Control group; 1=FPG; 2=HbA1c; 3=Fins; 4=hs-CRP; 5=IL-6; 6=TNF-α; 7=SBP; 8=DBP; 9=TC; 10=TG; 11=HDL-C; 12=LDL-C°.

### 3.3 Risk of bias assessment in the literature

**Figure 2:**
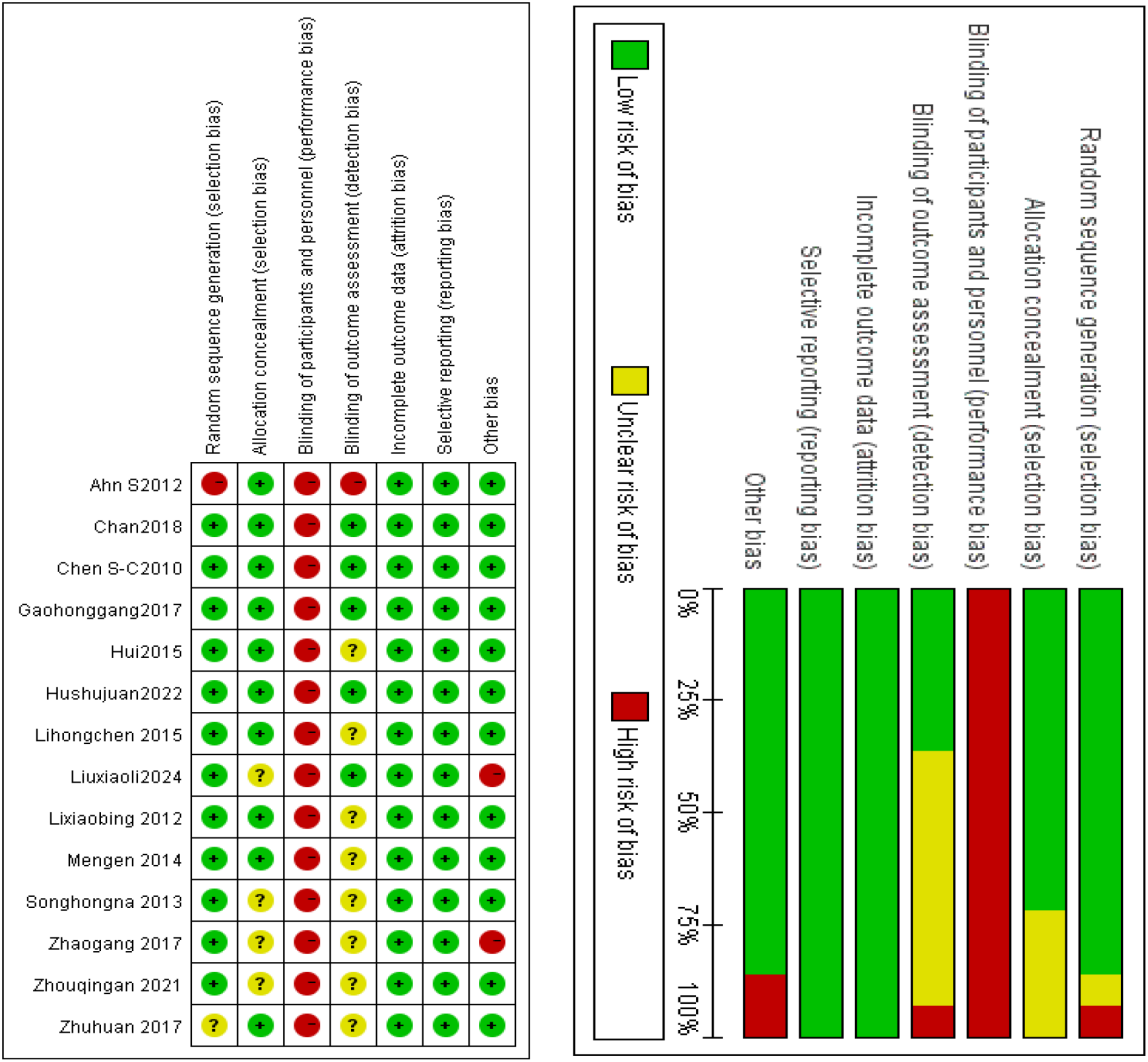
Assessment of risk of bias.

Two investigators (SYW. and LQZ) independently extracted methodological data from the Materials and Methods sections of all 14 studies. Risk of bias assessment: Methodological quality was appraised using the Cochrane Risk of Bias 2.0 (RoB 2.0) tool. Each domain was classified as low risk, high risk, or unclear risk. Discrepancy resolution: Conflicts in assessment were resolved through adjudication by a third investigator (XWQ). Results of bias assessment: Random sequence generation: Low risk (n = 12), unclear risk (n = 1), high risk (n = 1). Allocation concealment: Low risk (n = 10), unclear risk (n = 4). Blinding of participants/personnel: Not applicable (inherent impossibility due to Tai Chi’s physical intervention nature). Blinding of outcome assessment: Low risk (n = 4), high risk (n = 1), unclear risk (n = 9). Incomplete outcome data: Low risk (n = 14; no missing data). Selective reporting: Low risk (n = 14; all outcomes fully reported). Other biases: Low risk (n = 12), high risk (n = 2). **Figure 2** summarizes the risk-of-bias assessment results.

### 3.4 Meta-analysis results

#### 3.4.1 Fasting blood glucose

**Figure 3:**
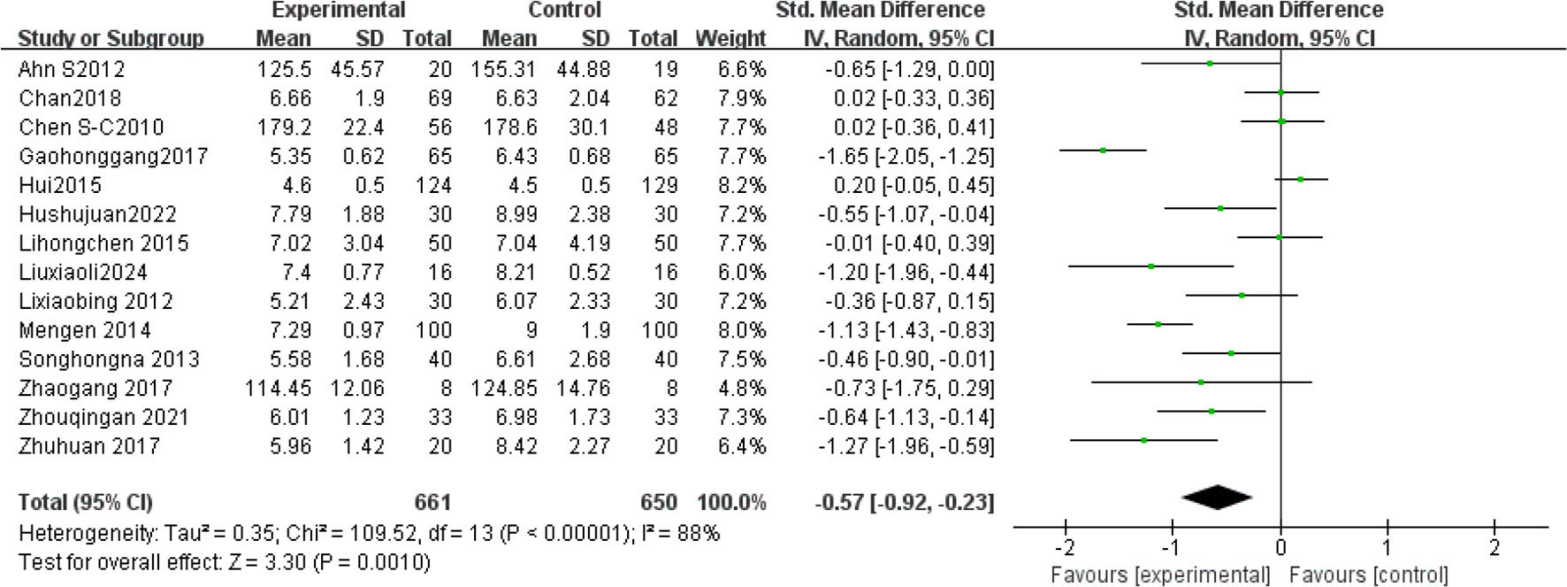
Meta-analysis of the effect of Tai Chi on FBG levels in T2DM patients.

Fourteen studies (1, 13, 23, 24, 32–41) analyzed FBG levels post-Tai Chi intervention in 1,311 participants. High heterogeneity was observed (I²= 88%, *P* < 0.00001), prompting use of a random-effects model. Pooled analysis demonstrated a significantly greater reduction in FBG levels in the Tai Chi group versus controls (SMD = -0.57, 95% CI [-0.92, -0.23], *P* = 0.001) (**Figure 3**).

(1) Sensitivity analysis

Sensitivity analysis revealed stable pooled effect sizes (SMD = -0.57, *P* = 0.001) upon sequential exclusion of individual studies (**Table 3**), confirming result robustness. However, heterogeneity persisted (I²> 75%), suggesting unmeasured confounding factors (e.g., intervention protocols, duration, frequency). Subgroup analyses were therefore conducted.

**Table 3:** The meta-analysis results after individual exclusion of studies.

Excluding studies	I^2^	SMD	95%CI	*P*
Ahn S 2012	89%	-0.57	[-0.93, -0.21]	0.002
Chan 2018	88%	-0.63	[-0.99, -0.26]	0.0008
Chen 2010	88%	-0.63	[-0.99, -0.26]	0.0007
Gao 2017	83%	-0.47	[-0.78, -0.17]	0.002
Hui 2015	84%	-0.64	[-0.97, -0.31]	0.0002
Hu 2022	89%	-0.58	[-0.94, -0.21]	0.002
Li 2015	89%	-0.62	[-0.99, -0.26]	0.0008
Liu 2024	89%	-0.53	[-0.89, -0.18]	0.003
Li 2012	89%	-0.59	[-0.96, -0.23]	0.001
Meng 2014	86%	-0.52	[-0.86, -0.18]	0.003
Song 2013	89%	-0.59	[-0.95, -0.22]	0.002
Zhao 2017	89%	-0.57	[-0.92, -0.21]	0.002
Zhou 2021	89%	-0.57	[-0.94, -0.21]	0.002
Zhu 2017	88%	-0.53	[-0.88, -0.18]	0.003

(2) Subgroup analysis

**Figure 4:**
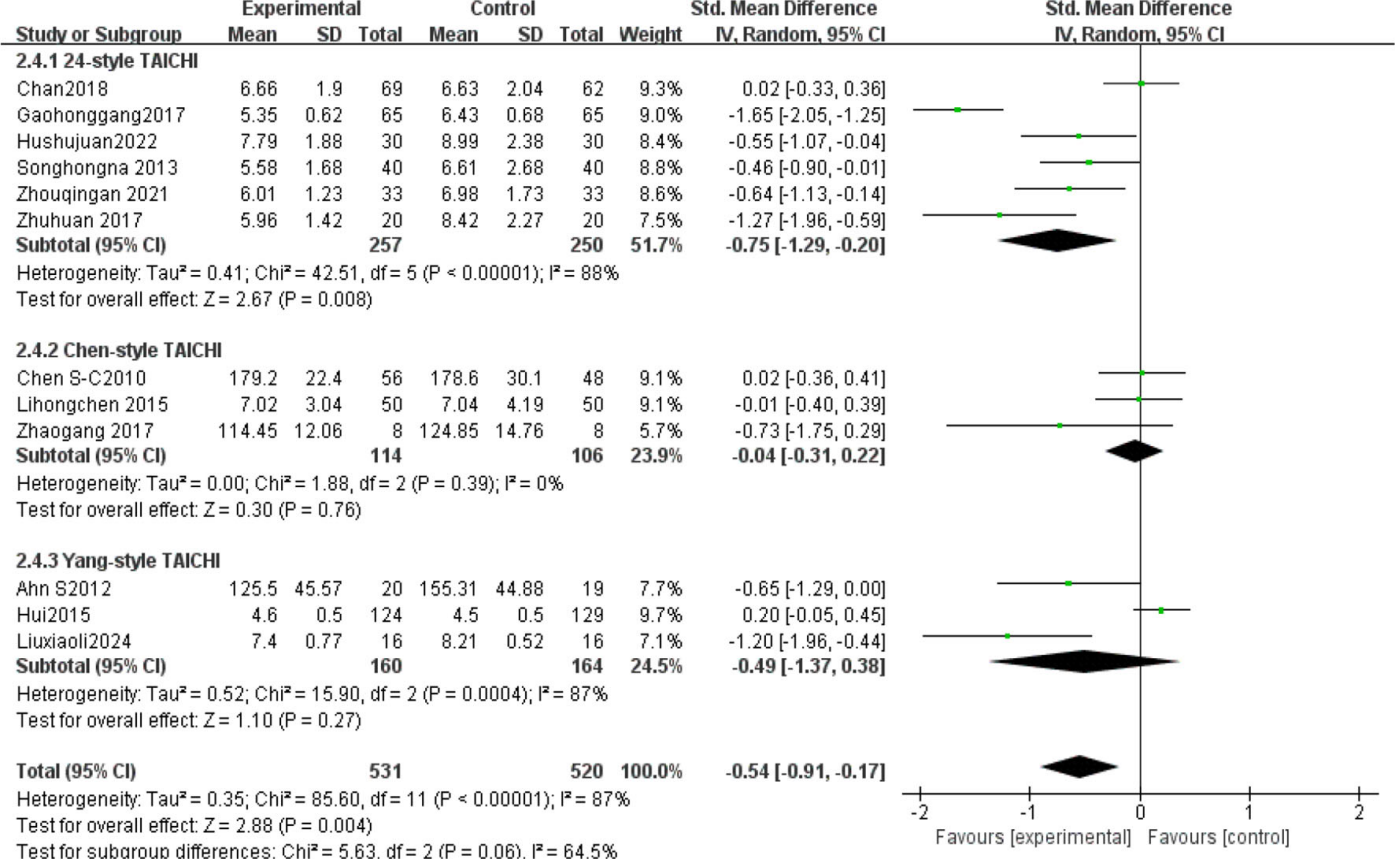
Subgroup analysis: style.

**Figure 5:**
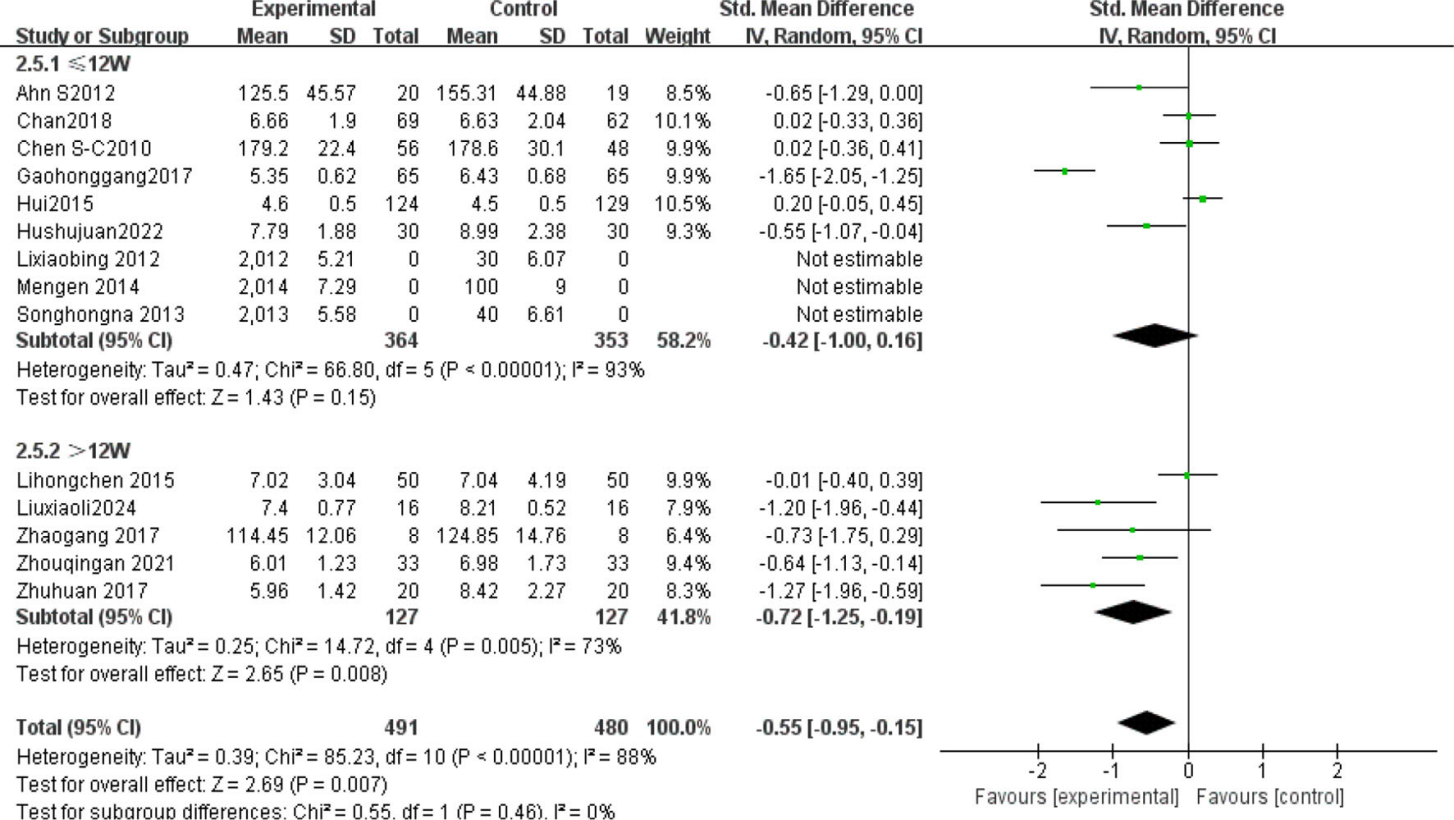
Subgroup analysis: intervention duration.

**Figure 6:**
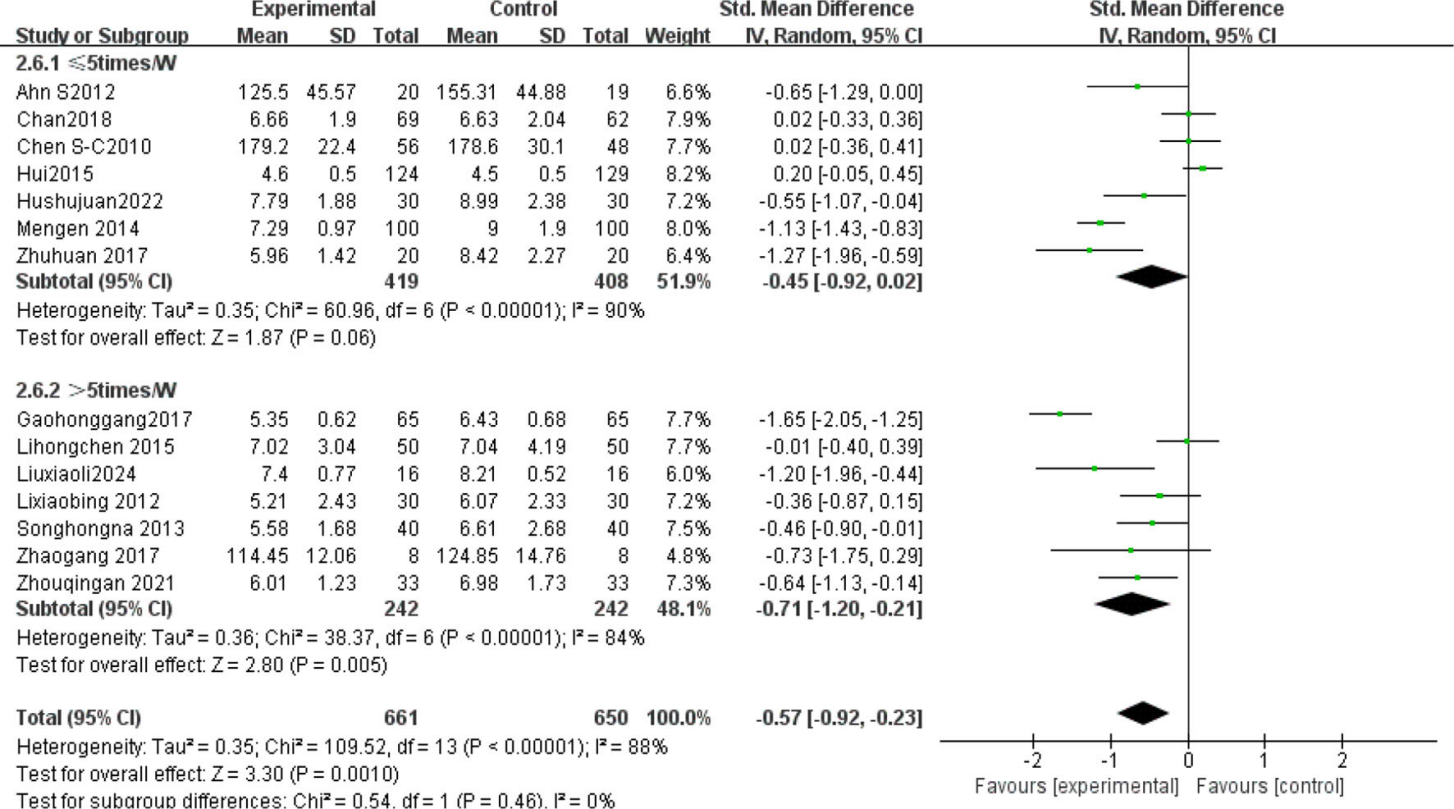
Subgroup analysis: intervention frequency.

**Figure 7:**
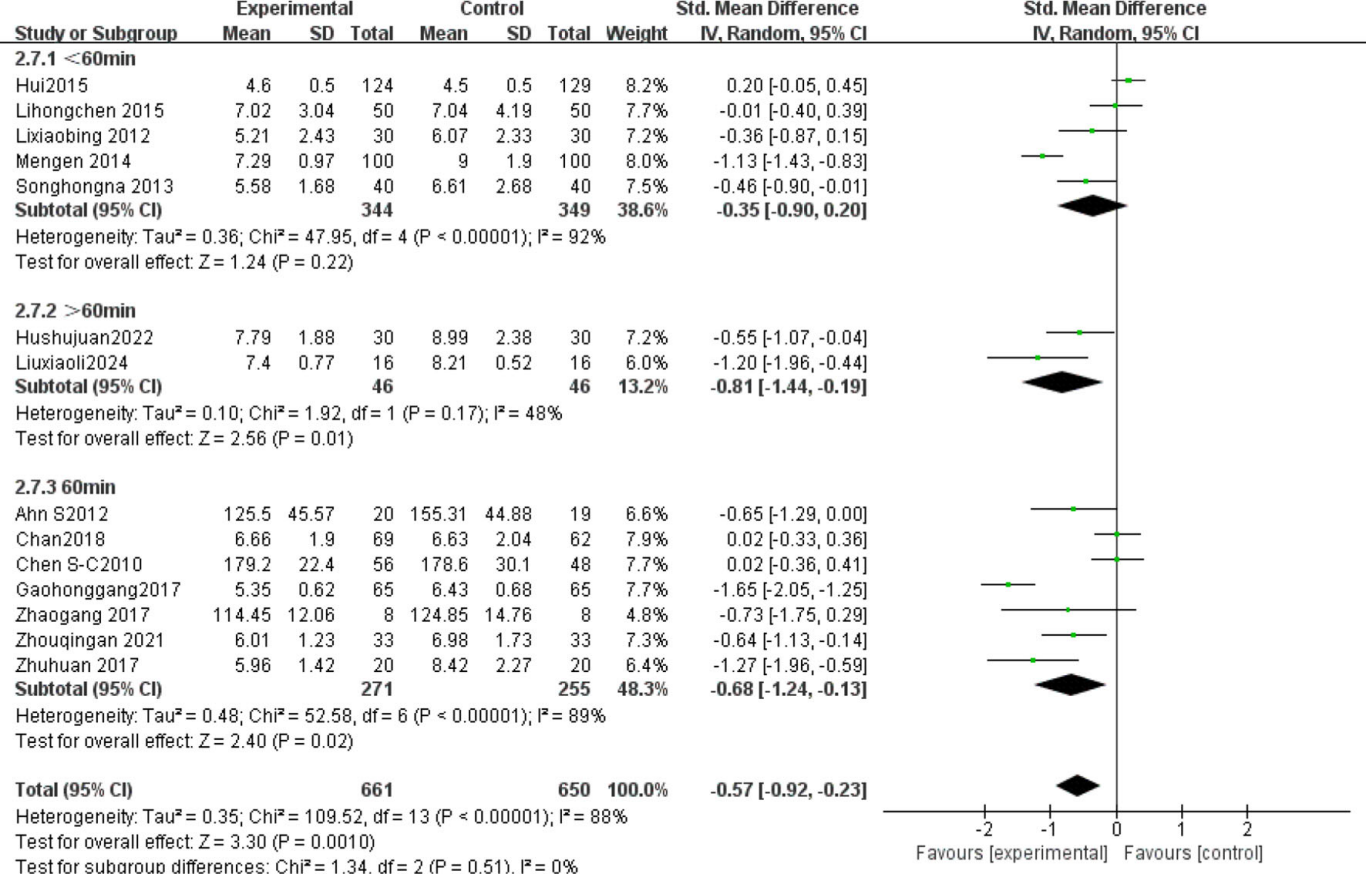
Subgroup analysis: daily exercise duration.

The subgroup analysis results: Tai Chi style (**Figure 4**): 24- style Tai Chi (n = 6 studies, 507 participants): *P* = 0.008. Chen-style Tai Chi (n = 3 studies, 220 participants): *P* = 0.76. Yang-style Tai Chi (n = 3 studies, 324 participants): *P* = 0.27. The overall effect was significant *P* =0.004, with the 24-form Tai Chi showing a more significant difference in improving FBG levels. Intervention duration (**Figure 5**): <12 weeks (n = 9 studies, 717 participants): P = 0.15 (non-significant). ≥12 weeks (n=5 studies, 254 participants): *P* = 0.008, indicating significant improvement in FBG levels for interventions lasting more than 12 weeks. Intervention frequency (**Figure 6**) :≤5 sessions/week (n = 7 studies, 827 participants): *P* = 0.06. >5 sessions/week (n = 7 studies, 484 participants): *P* = 0.005 (significant benefit), suggesting that an exercise frequency of more than 5 times per week was more effective in improving FBG levels. Daily Exercise Duration (**Figure 7**): <60 minutes (n = 5 studies, 693 participants): *P* = 0.22. >60 minutes (n = 2 studies, 92 participants): *P* = 0.01. 60 minutes (n = 7 studies, 526 participants): *P* = 0.02. Durations ≥60 minutes showed significant FBG improvement. The effect sizes and P-values for each subgroup are shown in **Table 4**.

**Table 4:** Subgroup analysis.

Subgroup	Number of studies	SMD	95%CI	*P*
Style				
24-style	6	-0.54	[-0.91, -0.17]	0.004
Chen-style	3			
Yang-style	3			
Intervention duration				
≤12 Weeks	9	-0.55	[-0.95, -0.15]	0.007
>12 Weeks	5			
Intervention frequency				
<≤5 sessions/week	7	-0.57	[-0.92, -0.23]	0.001
>5 sessions/week	7			
Session duration				
<60min	5	-0.57	[-0.92, -0.23]	0.001
>60min	2			
60min	7			

#### 3.4.2 HbA1c

**Figure 8:**
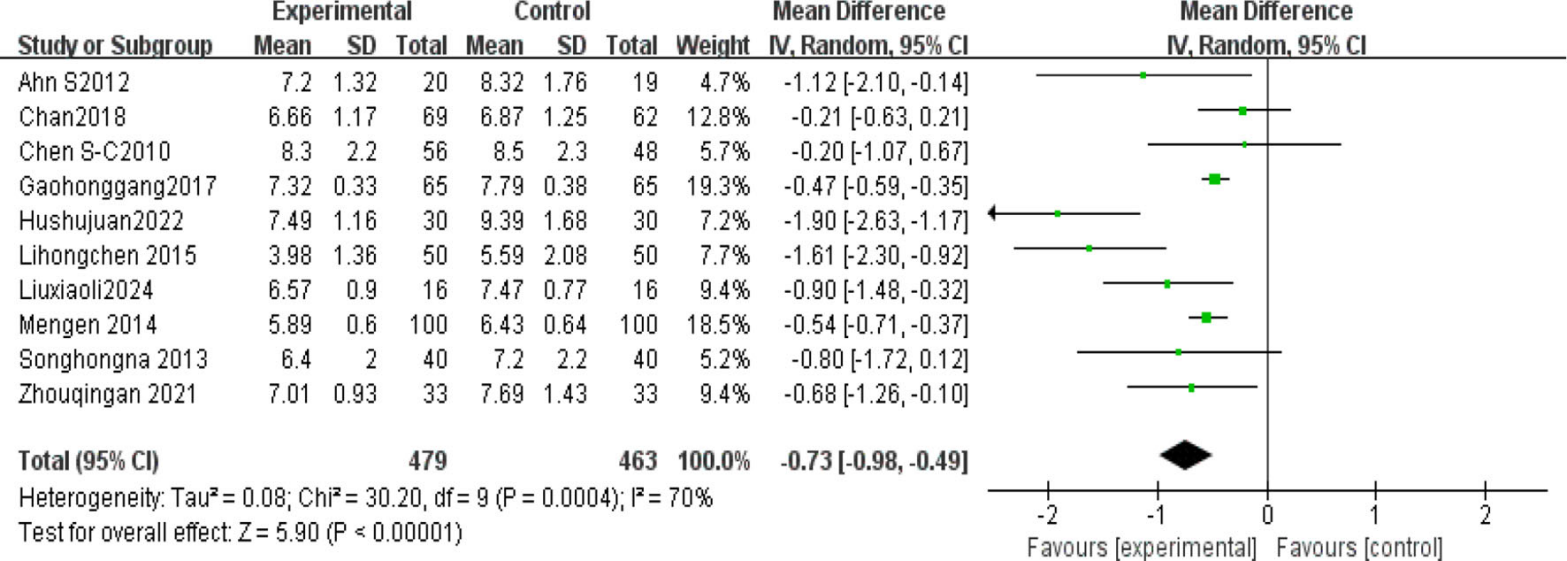
Meta-analysis of the effect of Tai Chi on HbA1c levels in T2DM patients.

Ten studies (4, 5, 19, 22–24, 36, 38–40) analyzed glycated hemoglobin (HbA1c) levels post-Tai Chi intervention in 942 participants. High heterogeneity was observed (I² = 70%), prompting use of a random-effects model. The Tai Chi group showed significant HbA1c reduction (MD = -0.73%, 95% CI [-0.98, -0.49], *P* < 0.00001) (**Figure 8**).

(2) Sensitivity analysis

The sensitivity analysis showed that after excluding the study by Hu(2022), heterogeneity decreased to a minimum I²=52%. The study involved 60 T2DM patients (30 in the experimental group and 30 in the control group) and showed significant baseline imbalance, as the experimental group had a lower baseline HbA1c (8.07) compared to the control group (9.16). Additionally, the control group showed a larger standard deviation in HbA1c(1.59% to 1.68%), suggesting high data variability. However, the results of the study *P <*0.00001 were stable and showed a significant difference **Table 5**.

**Table 5:** The meta-analysis results after individual exclusion of studies.

Excluding studies	I^2^	MD	95%CI	*P*
Ahn S 2012	72%	-0.71	[-0.97, -0.46]	<0.00001
Chan 2018	71%	-0.81	[-1.08, -0.55]	<0.00001
Chen 2010	73%	-0.77	[-1.02, -0.52]	<0.00001
Gao 2017	71%	-0.83	[-1.18, -0.49]	<0.00001
Hu 2022	52%	-0.60	[-0.80, -0.41]	<0.00001
Li 2015	62%	-0.64	[-0.86, -0.42]	<0.00001
Liu 2024	72%	-0.72	[-0.98, -0.46]	<0.00001
Meng 2014	74%	-0.82	[-1.17, -0.48]	<0.00001
Song 2013	73%	-0.73	[-0.99, -0.48]	<0.00001
Zhou 2021	73%	-0.75	[-1.01, -0.48]	<0.00001

#### 3.4.3 Blood lipids

**Figure 9:**
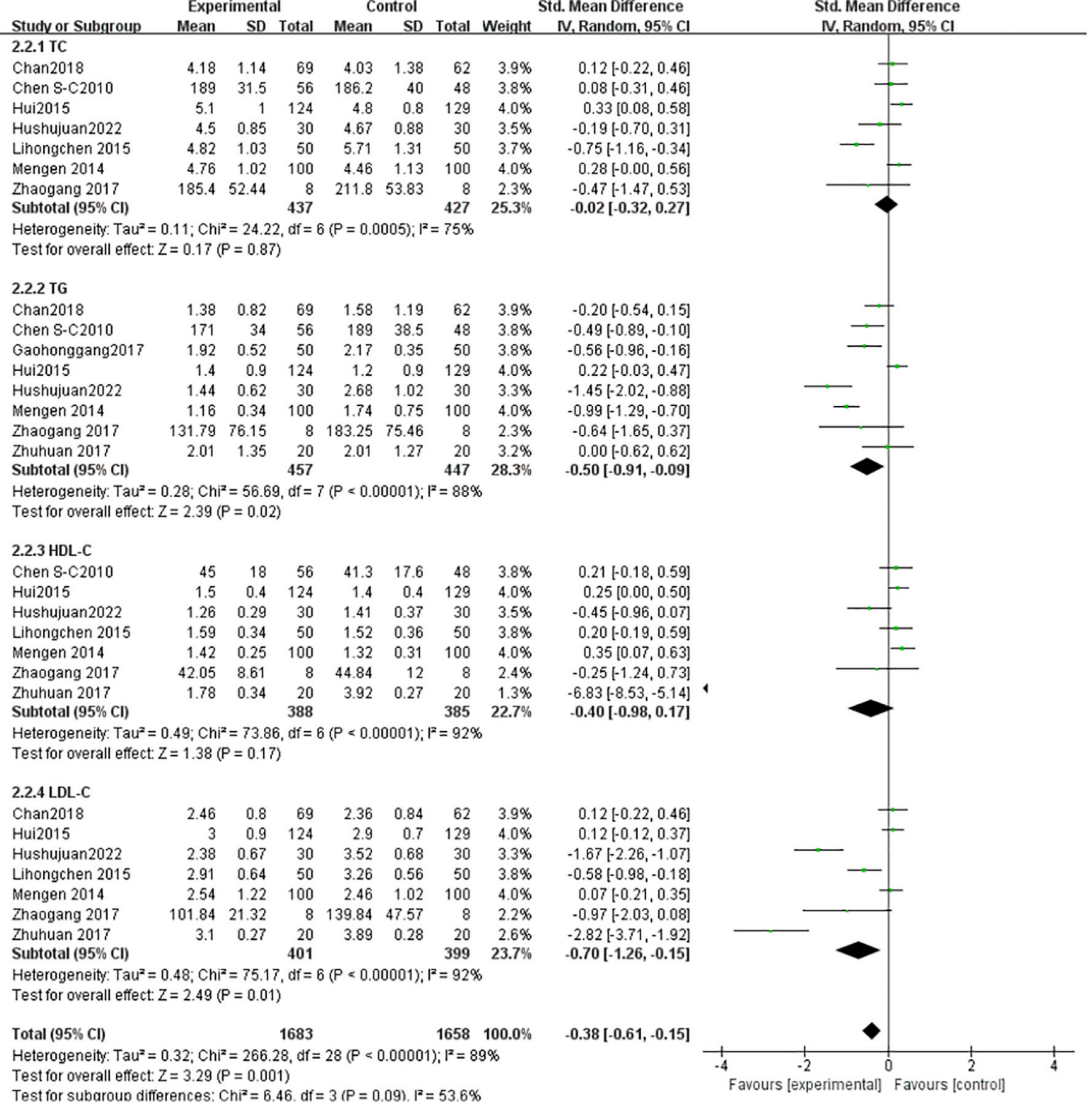
Meta-analysis of the effect of Tai Chi on Blood lipids in T2DM patients.

TC: Seven studies (1, 13, 24, 33–35, 38) (N = 964) showed high heterogeneity (I²= 75%), analyzed via random-effects model (SMD = -0.02, 95% CI [-0.32, 0.27], *P* = 0.87; non-significant). TG: Eight studies (1, 13, 23, 33–35, 38, 41) (N = 907) exhibited high heterogeneity (I² = 88%), analyzed via random-effects model (SMD = -0.50, 95% CI [-0.91, -0.09], *P* = 0.02). HDL-C: Seven studies (1, 13, 24, 34, 35, 38, 41) (N = 773) showed high heterogeneity (I² = 92%), analyzed via random-effects model (SMD = -0.40, 95% CI [-0.98, 0.17], *P* = 0.17). LDL-C: Seven studies (1, 13, 24, 33, 35, 38, 41) (N = 800) showed high heterogeneity (I² = 92%), analyzed via random-effects model (SMD = -0.70, 95% CI [-1.26, -0.15], *P* = 0.01) (**Figure 9**).

#### 3.4.4 Blood pressure

**Figure 10:**
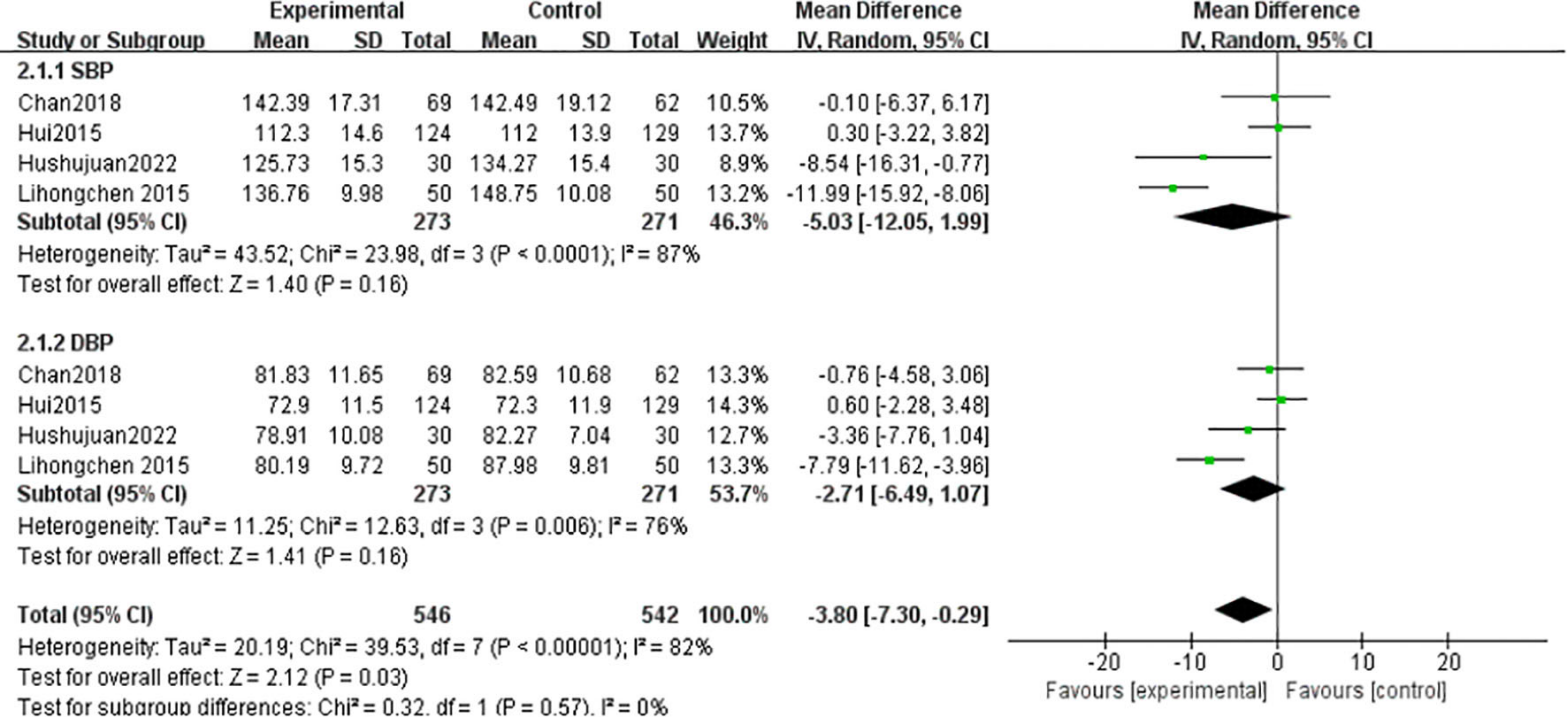
Meta-analysis of the effect of Tai Chi on Blood pressure in T2DM patients.

SBP: Four studies (1, 24, 33, 35) (n = 544) showed high heterogeneity (I² = 87%), analyzed via random-effects model (MD = -5.03 mmHg, 95% CI [-12.05, 1.99], *P* = 0.16; non-significant). DBP: Heterogeneity (I²= 82%) prompted random-effects analysis (MD=-3.80 mmHg, 95%CI[-7.30,-0.29], *P*=0.16) (**Figure 10**).

#### 3.4.5 Inflammatory factors

**Figure 11:**
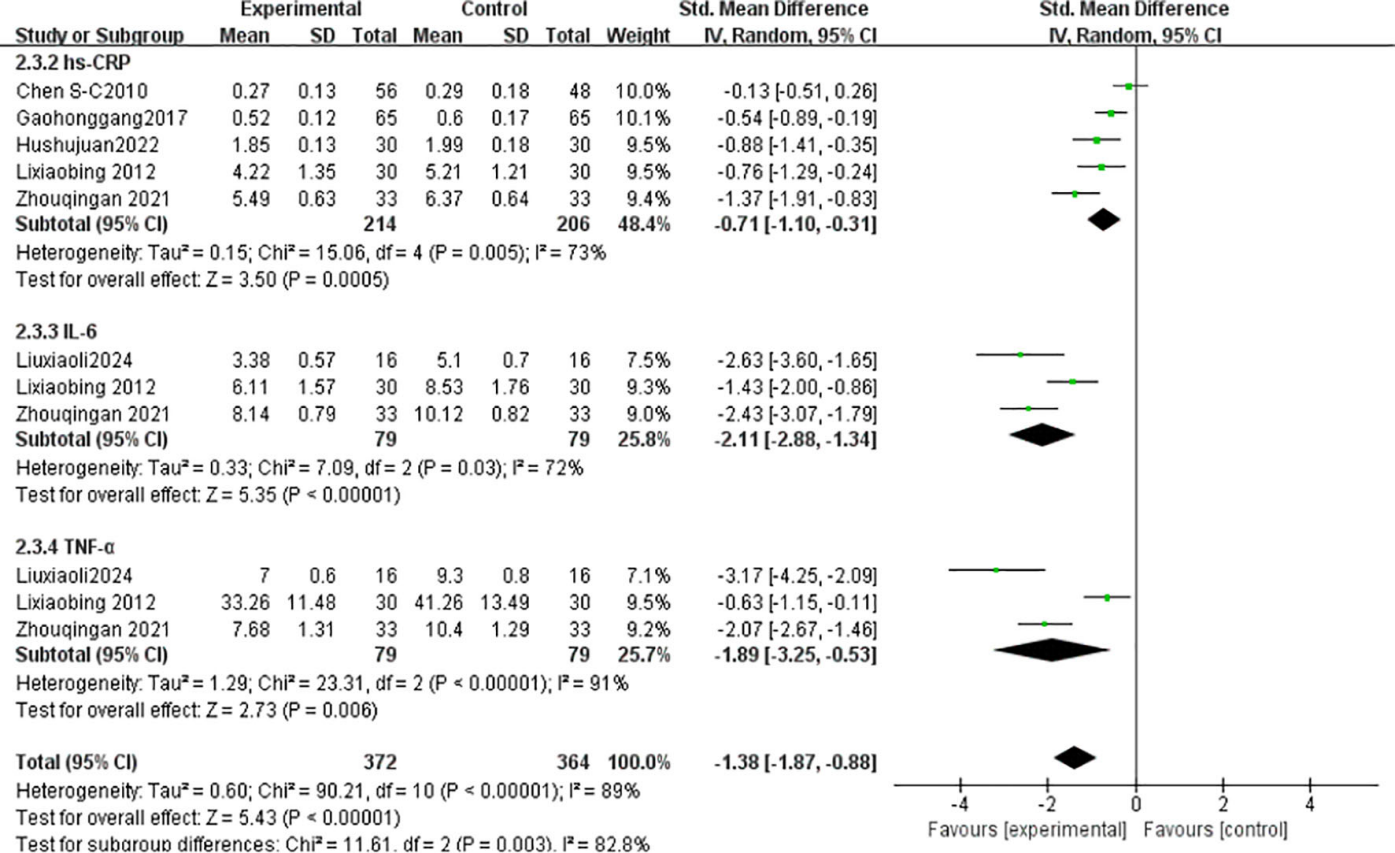
Meta-analysis of the effect of Tai Chi on Inflammatory factors in T2DM patients.

hs-CRP: Five studies (23, 34, 35, 37, 40) (n = 420) showed high heterogeneity (I² = 73%), analyzed via random-effects model (SMD = -0.71, 95% CI [-1.10, -0.31], *P* = 0.0005). IL-6: Three studies (36, 37, 40) (n = 158) exhibited high heterogeneity (I² = 72%), analyzed via random-effects model (SMD = -2.11, 95% CI [-2.88, -1.34], *P* < 0.00001). TNF-α: Three studies (36, 37, 40) (n = 158) showed high heterogeneity (I² = 91%), analyzed via random-effects model (SMD = -3.25, 95% CI [-3.25, -0.53], *P* = 0.006) (**Figure 11**).

### 3.5 GRADE evidence quality evaluation

Through the GRADE system for evidence quality analysis, Tai Chi has shown significant effects and sufficient evidence in improving blood glucose-related indicators FPG,HbA1c,Fins compared to other therapies, while more high-quality studies are needed to support its effects on blood pressure, blood lipids, and other indicators. It is recommended to prioritize critical indicators and optimize bias control in subsequent research. Among them, the evidence quality for HbA1c is “high,” while the evidence for FBG, Fins, blood pressure, blood lipids, and inflammatory factors is “moderate” (**Table 6**).

**Table 6:** GRADE evidence quality classification.

Certainty assessment							№ of patients		Effect		Certainty	Importance
№ of studies	Study design	Risk of bias	Inconsistency	Indirectness	Imprecision	Other considerations	TAICHI	comparison	Relative (95% CI)	Absolute (95% CI)		
FPG												
13	randomized trials	serious^a^	not serious	not serious	not serious	none	641	630	–	SMD 0.53 lower (0.88 lower to 0.18 lower)	⨁⨁⨁◯ Moderate^a^	CRITICAL
HbA1c												
10	randomized trials	not serious^a^	not serious	not serious	not serious	none	479	463	–	MD 0.73 lower (0.98 lower to 0.49 lower)	⨁⨁⨁⨁ High^a^	CRITICAL
Fins												
6	randomized trials	serious^a^	not serious	not serious	not serious	none	234	234	–	SMD 0.48 lower (0.82 lower to 0.14 lower)	⨁⨁⨁◯ Moderate^a^	CRITICAL
Blood pressure												
8	randomized trials	serious^a^	not serious	not serious	not serious	none	546	542	–	MD 3.8 lower (7.3 lower to 0.29 lower)	⨁⨁⨁◯ Moderate^a^	IMPORTANT
Blood lipid												
9	randomized trials	serious^a^	not serious	not serious	not serious	none	1683	1658	–	SMD 0.38 lower (0.61 lower to 0.15 lower)	⨁⨁⨁◯ Moderate^a^	IMPORTANT
Inflammatory factor												
6	randomized trials	serious^a^	not serious	not serious	not serious	none	372	364	–	SMD 1.38 lower (1.87 lower to 0.88 lower)	⨁⨁⨁◯ Moderate^a^	IMPORTANT

CI, confidence interval; MD, mean difference; SMD, standardized mean difference; ^a^There was high heterogeneity in this study.

## 4 Discussion

### 4.1 Interpretation of meta-analysis results

This meta-analysis aimed to quantify Tai Chi’s effects on metabolic/inflammatory markers in T2DM. Key findings include: (1) significant reductions in FBG, HbA1c, TG, LDL-C, and inflammatory markers; (2) optimal FBG improvement with 24-style Tai Chi, ≥12-week duration, >5 sessions/week, and ≥60-min sessions; (3) non-significant effects on SBP and HDL-C.

#### 4.1.1 FBG

In single-arm meta-analyses of continuous outcomes, data synthesis poses methodological challenges. Although combining pre- and post-intervention measurements from individual studies can help assess intervention effects, this method may not entirely eliminate bias. Our meta-analysis pooled data from 14 RCTs involving 1,311 patients with T2DM. Tai Chi significantly reduced fasting blood glucose (FBG) versus controls (SMD= -0.57, 95% CI [-0.92, -0.23], *P* = 0.001), demonstrating a medium effect size (Cohen’s d = 0.57).This finding is consistent with previous studies (42, 43), confirming Tai Chi’s superior FBG-lowering efficacy over conventional therapies in T2DM. Substantial heterogeneity (I² = 88%) necessitated sensitivity and subgroup analyses to identify moderators and assess clinical relevance. Sensitivity analysis confirmed result robustness despite persistent heterogeneity (I² > 75% post-exclusion). Subgroup analyses stratified by Tai Chi style, intervention duration (weeks), frequency (sessions/week), and Daily Exercise Duration (minutes) were conducted. The 24-style Tai Chi subgroup showed the largest FBG reduction (*P* < 0.01 vs. other styles). This corroborates Xun (42), whose systematic review identified the 24-form Tai Chi as optimal for FBG reduction in diabetes management. Subgroup analyses revealed that, compared to other Tai Chi styles (such as Chen and Yang), the 24-form TaiChi demonstrated a greater SMD in FBG improvement, which was statistically significant P<0.01. Xun attributed this superiority to the 24-form’s standardized movement sequences, which enhance long-term adherence. Intervention Duration: Interventions ≥12 weeks yielded clinically meaningful FBG reductions (SMD = -0.85, *P* < 0.0001). Similarly, Xun’s (42) research further validated that the 24-form Tai Chi has the most significant effect on FBG improvement, with stable effects when the intervention duration is ≥12 weeks. A meta-analysis by Su (44) showed that when Tai Chi interventions lasted ≥12 weeks, the reduction in FBG was more pronounced (SMD=−0.85, *P <*0.0001), and long-term interventions (e.g.,24weeks) had better effects on HbA1c. This may be related to a mechanism of dose-dependency, where short-term interventions may only affect acute glucose regulation, while long-term interventions (>12weeks) can result in cumulative effects by improving insulin sensitivity and microcirculation (45). Intervention Frequency: Studies have shown that an exercise frequency of more than 5 times per week is more effective in improving FBG. Peng Yong’s (46) research showed that practicing Tai Chi six times per week (1hour each time) significantly reduced FBG and HbA1c P<0.05, with the mechanism related to improving vascular endothelial function (e.g., increased NO, eNOS levels) and enhancing microcirculatory reactivity. High-frequency exercise may improve glucose metabolism by continuously stimulating insulin receptor activity (e.g., increasing the number of low−affinity receptors). In contrast, a combination of four Tai Chi sessions per week and two resistance training sessions improved FBG but was less effective than the high-frequency TaiChi group 6 times per week. This might be due to the differing metabolic pathways of resistance training (such as preferentially consuming muscle glycogen rather than liver glycogen), which diminishes the synergistic effect of high-frequency aerobic exercise. Daily Exercise Duration: Daily exercise durations of 60 minutes or more showed a more significant improvement in FBG, with both groups showing statistically significant differences (*P <*0.05). Xia’s (47) study found that when the total duration of Tai Chi intervention exceeded 3 months and daily sessions were ≥60 minutes, the effect on lowering FBG was more significant (SMD=−0.93, *P <*0.001). Similarly, Zhang Jianwei’s study48showed that T2DM patients practicing 60 minutes of Tai Chi 5days per week, for 8 weeks significantly reduced their FBG, HbA1c, and insulin resistance index (HOMA−IR), with male patients showing a significantly higher improvement in FBG than female patients (P<0.05). The study suggested that a longer duration (per session 60minutes) might enhance insulin sensitivity and regulate glucose and lipid metabolism pathways.

#### 4.1.2 HbA1c

HbA1c is a stable product formed through a non-enzymatic glycation reaction between glucose in the blood and the N-terminal valine of hemoglobin’s β-chain. Its concentration correlates with blood glucose levels and reflects the average blood glucose level over the past 8–12 weeks (48). Since 2010, it has been included as one of the core diagnostic criteria for diabetes by the ADA and WHO (diagnostic threshold≥6.5) (48). The results showed I²=70%, thus sensitivity analysis was conducted. After excluding Hu (2022), heterogeneity decreased to a minimum (I²=52%), MD=0.60, 95% CI[0.80,−0.41], *P* < 0.00001. We identified sources of heterogeneity, such as baseline imbalance and high data variability. However, the results remained stable, with a statistically significant difference P<0.00001. Zhou (49) included 23 studies(1235 patients), with most interventions lasting more than 3 months, and incorporating various Tai Chi styles. The study showed that Tai Chi significantly reduced HbA1c(MD=−0.88, *P*=0.002), and also improved FPG and insulin resistance (HOMA−IR). Additionally, the study emphasized the cumulative effect of long-term interventions on metabolic indicators. Similarly, Chao (50) reported high heterogeneity in effect size I²=70, but their conclusion differed from this study: Tai Chi’s effect on HbA1c reduction (MD=−0.21) was not statistically significant(*P* =0.31). Chen (34) found no significant reduction in HbA1c after 12 weeks of Tai Chi intervention (*P* =0.064), although improvements in BMI and blood lipids were significant. This may be due to the small sample size (n=40), and a longer intervention period might be needed to affect HbA1c. This also underscores the importance of long-term interventions in improving insulin sensitivity and achieving cumulative effects for blood glucose regulation (45).

#### 4.1.3 Blood Lipids

Dyslipidemia is an independent risk factor for diabetes. Tao Ran (51), mentioned that dyslipidemia (high TG, low HDL−C) increases the risk of diabetes by 2.4 times (OR=2.40). Each standard deviation increase in TG raises the risk of diabetes by 26% (OR=1.26). Guo’s (52) meta-analysis included 26 studies (n=1779) and found that Tai Chi significantly reduced TC (MD=−0.24,95 CI [-0.58, 0.10]), but this did not reach statistical significance P>0.05. In line with this study’s findings, there was no significant difference in TC reduction between the Tai Chi and control groups. Tang Qing’s (26) study showed that Tai Chi significantly reduced TG(MD=-0.33, 95% CI [-0.49, -0.17], *P <*0.001), likely related to exercise-induced fat metabolism, indicating that Tai Chi can improve lipid oxidation. This finding is consistent with this study’s result(SMD=-0.50, *P* =0.02). Yan (53) included 4 RCTs and 5 non-RCTs, finding no significant improvement in HDL-C with Tai Chi(*P* =0.12). In this study, the SMD = -0.40, *P* = 0.17, indicating no significant difference in HDL-C improvement between the Tai Chi and control groups. This may be because HDL-C requires a longer time to improve (20weeks), which short-term studies may not capture (38).Guo (52) found a significant reduction in LDL-C(MD=-0.32, 95% CI [-0.59, -0.05], *P <*0.05), attributed to Tai Chi’s ability to reduce oxidative stress and inflammation, consistent with the results of this study.

#### 4.1.4 Blood pressure

Hypertension increases oxidative stress, leading to endothelial dysfunction, exacerbating insulin resistance, and consequently raising blood glucose levels (54). This study’s results align with previous research (50), showing that Tai Chi did not achieve statistical significance in its combined effect on blood pressure indicators (SBP: MD = -1.39, 95% CI [-1.95, -0.84]; DBP: MD = -0.50, 95% CI [-1.02, 0.02]). This indicates that Tai Chi may not effectively lower blood pressure in T2DM patients. The reason could be that metabolic disorders and vascular complications in T2DM patients may reduce the blood pressure-lowering effect of Tai Chi compared to patients with only hypertension.

#### 4.1.5 Inflammatory factors

Inflammatory factors such as IL-6, TNF-α, and hs-CRP play a role in T2DM by promoting insulin resistance (IR) and β-cell dysfunction. Studies have shown that IL-6 activates the JAK/STAT pathway, inhibiting insulin receptor substrate (IRS) phosphorylation and hindering insulin signal transduction (55); TNF-α downregulates GLUT4 expression, interfering with glucose metabolism. In T2DM patients, the levels of these pro-inflammatory factors are significantly elevated and positively correlated with HbA1c and fasting blood glucose (56). Additionally, research indicates that insulin resistance is closely related to chronic inflammation(increased IL−6 and TNF−αlevels), and Tai Chi may indirectly suppress inflammation by improving metabolic disorders (57). A 12-month Tai Chi intervention significantly reduced IL-6 and hs-CRP levels in middle-aged and elderly T2DM patients (*P <*0.01), and also improved blood lipids and arterial stiffness. Tsang’s (58) randomized, double-blind controlled trial showed no significant improvement in insulin sensitivity (HOMA2−IR) and HbA1c levels in the Tai Chi group, and no significant changes in inflammatory factors (e.g.,IL−6,TNF−α). The authors suggested that Tai Chi’s intensity might not be sufficient to induce significant metabolic and inflammatory improvements, particularly for patients with obesity or severe insulin resistance. Studies have highlighted the importance of long-term adherence to Tai Chi for inflammation suppression (50), further supporting the relationship between inflammation factor improvement and intervention duration. Notably, a study involving 66 T2DM patients with peripheral vascular disease found that 12 weeks of Tai Chi significantly reduced serum TNF-α, IL-6, and hs-CRP levels (P<0.05), with better results than medication alone40. Although this study was not a meta-analysis, its high-quality RCT provides direct evidence for the improvement of inflammatory factors, and the inflammatory indicators analyzed align with our study’s findings.

### 4.2 Biological mechanisms of Tai Chi in treating T2DM patients

Improving Insulin Sensitivity and Regulating Insulin Signaling Pathways. Firstly, Tai Chi improves insulin sensitivity by regulating the balance of the autonomic nervous system. Its calming exercise mode significantly reduces sympathetic nervous tension and decreases the secretion of catecholamines (such as adrenaline and noradrenaline) (59), thereby alleviating the inhibitory effect of β-adrenergic receptors on the insulin signaling pathway. Heart rate variability (HRV) analysis shows that long-term Tai Chi intervention enhances parasympathetic nervous activity, improves insulin receptor (IR) tyrosine kinase activity, and promotes tyrosine phosphorylation of insulin receptor substrate-1 (IRS−1) (60). This process activates the downstream PI3K/Akt signaling pathway, driving the translocation of glucose transporter 4 (GLUT4) to the cell membrane, which increases glucose uptake by skeletal muscle and adipose tissue by 15-20% (61). Secondly, Tai Chi’s regulation of metabolic pathways has multi-target characteristics. Studies have found that Tai Chi can activate the AMPK pathway, improve mitochondrial function, and promote GLUT4 mRNA expression (60), while reducing ROS levels to decrease the abnormal degradation of insulin signaling molecules by the ubiquitin-proteasome system (62). Clinical evidence shows that 12 weeks of Tai Chi intervention resulted in a 0.76% reduction in HbA1c and a 0.69 reduction in HOMA-IR index (44), with particularly significant effects on insulin sensitivity from low-platform Tai Chi. These mechanisms together form a systemic regulatory network that improves glucose metabolism in T2DM patients through Tai Chi.

Protecting β-Cell Function. In terms of oxidative stress regulation, chronic hyperglycemia activates NADPH oxidase to promote reactive oxygen species (ROS) generation, leading to a decrease in the mitochondrial membrane potential of β-cells and activating the Caspase-3-dependent apoptosis pathway. Tai Chi intervention significantly alleviates oxidative damage by reducing blood glucose fluctuations with HbA1c decreasing by 0.5−1.2% (63). Secondly, Tai Chi influences insulin secretion by regulating autonomic nervous balance. Vagal nerve stimulation activates the β-cell M3 receptor through acetylcholine, triggering the phospholipase C-IP3 signaling pathway, promoting the release of calcium ions from the endoplasmic reticulum, and enhancing glucose-stimulated insulin secretion (GSIS) (64). Meanwhile, vagal nerve activity inhibits the secretion of glucagon from α-cells, forming a dynamic balance of the “insulin-glucagon axis” (64). It is worth noting that the moderate exercise intensity of Tai Chi(50-69% Max heart rate) avoids the cortisol level increase that can occur with intense exercise approximately(About a 12% decline) (65), thereby reducing the metabolic load on β-cells. Future research should further clarify the relationship between Tai Chi intervention dosage and β-cell regeneration potential, and explore its synergistic effects with traditional hypoglycemic drugs.

Improving Endothelial Function. Tai Chi significantly enhances the bioavailability of nitric oxide (NO), thereby improving flow-mediated vasodilation (FMD increases by 2.1) and inhibiting the formation of atherosclerotic plaques. This mechanism has been supported by multiple studies. For example, some studies indicate that Tai Chi can regulate endothelial cell function, reduce the expression of inflammatory mediators, and improve arterial stiffness and glucose-lipid metabolism (66). In addition, Tai Chi can reduce arterial stiffness by increasing endothelium-dependent vasodilation, further optimizing cardiovascular health (67).

In conclusion, Tai Chi improves T2DM management through three interconnected pathways:(1) Insulin Sensitivity Enhancement: Modulates autonomic balance, activates PI3K/Akt signaling, and upregulates GLUT4 translocation.(2) β-Cell Protection: Reduces oxidative stress and cortisol-mediated apoptosis while enhancing glucose-stimulated insulin secretion.(3) Endothelial Function Improvement: Boosts NO bioavailability, suppressing inflammation and arterial stiffness.

### 4.3 Application value of Tai Chi in preventing and treating diabetes

Tai Chi combines meditation with exercise, making it highly acceptable psychologically and culturally, especially among Asian populations. The integration of “Internet + Tai Chi” remote guidance models can further enhance the accessibility of diabetes management in remote areas (43). It is noteworthy that Tai Chi, as a community-based exercise, not only helps improve patients’ blood glucose control and metabolic indicators but also enhances their quality of life. For instance, Tai Chi interventions can significantly improve patients’ HbA1c levels, fasting blood glucose, and blood lipid indicators, while reducing anxiety and depressive symptoms (68). Additionally, Tai Chi can enhance muscle strength and improve cardiopulmonary function, thereby improving patients’ overall health (24).

Unique Advantages of Non-Drug Interventions. Studies show that Tai Chi’s gentle movements and controllable intensity avoid the hypoglycemic risks associated with high-intensity exercise. For example, research shows that the incidence of adverse events (e.g., joint injuries, hypoglycemia) in the Tai Chi intervention group was significantly lower than that in the aerobic exercise group(3.2% vs. 8.7%) (43) Furthermore, Tai Chi can accommodate people with different physical conditions, including those with prediabetes, diagnosed T2DM, and comorbidities. For example, for T2DM patients with diabetic peripheral neuropathy (DPN), Tai Chi’s balance training not only reduces the risk of falls but also improves foot microcirculation (35).

In summary, Tai Chi provides a comprehensive and effective health management approach for diabetes patients. Future research should further explore the long-term effects of Tai Chi on diabetes complications and its potential molecular mechanisms to better guide clinical practice.

### 4.4 Strengths and limitations

This study expands the evidence base by integrating multiple metabolic and inflammatory outcomes (e.g., FBG, HbA1c, blood pressure, lipids, hs-CRP, IL-6, TNF-α), addressing the narrow biomarker scope of prior Tai Chi research in T2DM.

Limitations: Selection bias risk: Despite systematic searches across six databases and duplicate screening, potential omission of relevant studies cannot be fully excluded due to database selection constraints. High heterogeneity: Unblinded study designs (inherent to Tai Chi interventions) and variability in intervention protocols (e.g., styles, durations) may contribute to residual heterogeneity, necessitating cautious interpretation. Inflammatory biomarker scarcity: The paucity of RCTs measuring inflammatory markers (hs-CRP, IL-6, TNF-α) limits causal inferences. Future trials should prioritize inflammatory pathway analyses. Recommendations: Standardized RCT protocols with blinding of outcome assessors. Consolidated core outcome sets (e.g., HbA1c, HOMA-IR, hs-CRP). Longitudinal designs (>12 months) to assess durability and safety.

### 4.5 Future research suggestions

1. Expand Sample Size and Diversify Population: It is recommended to include a larger and more diverse cohort of individuals with T2DM, encompassing varying ages, disease durations, geographical locations, and cultural backgrounds. This will enhance the generalizability of the findings, reduce statistical errors associated with smaller samples, and strengthen the reliability of the conclusions. 2. Extend Intervention Duration: While current studies predominantly feature intervention periods of 12–24 weeks, future research should implement longer-term follow-up e.g.,1−3years. This is essential to observe the sustained effects of Tai Chi on glycemic control and to assess its potential for preventing diabetes-related complications.3. Standardize Tai Chi Intervention: To minimize intervention heterogeneity, future studies should standardize the Tai Chi style e.g., Chen−style, Yang−style, movement protocols, practice intensity e.g., frequency per week, duration per session, and teaching procedures. Combine with Other Interventions: Research should explore the synergistic effects of Tai Chi when combined with dietary control, pharmacotherapy, or other exercise modalities e.g., walking, yoga on glycemic management. This will provide clinicians with a broader range of effective intervention options.4. Explore Physiological and Molecular Mechanisms: Future work should utilize biomarker assessments e.g., insulin sensitivity, inflammatory, gut microbiota to elucidate the biological mechanisms through which Tai Chi regulates blood glucose, thereby providing a theoretical foundation for its effects.

## 5 Conclusion

Tai Chi significantly improves FBG, HbA1c, TG, LDL-C, and inflammatory markers (hs-CRP, IL-6, TNF-α) in T2DM patients, but not BP or HDL-C. For optimal FBG reduction, clinicians should recommend 24-style Tai Chi, practiced ≥5 times/week for ≥60 min/session over ≥12 weeks. These findings support Tai Chi as an effective adjunct therapy for global metabolic and inflammatory management in T2DM.

## Data Availability

The raw data supporting the conclusions of this article will be made available by the authors, without undue reservation.
